# Clinical predictors for surfactant retreatment in preterm infants with respiratory distress syndrome: the results of a pooled analysis

**DOI:** 10.1186/s13052-024-01828-1

**Published:** 2025-01-05

**Authors:** Carlo Dani, Chiara Poggi, Massimo Agosti, Massimo Bellettato, Pasqua Betta, Paolo Biban, Luigi Corvaglia, Raffaele Falsaperla, Carlo Forcellini, Diego Gazzolo, Eloisa Gitto, Camilla Gizzi, Paola Lago, Gianluca Lista, Gianfranco Maffei, Fabio Mosca, Marcello Napolitano, Gianfranco Scarpelli, Fabrizio Sandri, Daniele Trevisanuto, Giovanni Vento, Iuri Corsini, Simone Pratesi, Luca Boni

**Affiliations:** 1 Department of Neurosciences, Psychology, Drug Research and Child Health, University of Florence, Florence, Italy; 2 Division of Neonatology, Careggi University Hospital of Florence, Florence, Italy; 3 Maternal and Child Health Department, Del Ponte Hospital, A.O. Di Circolo Fondazione Macchi, Varese, Italy; 4 Division of Neonatology, Ospedale San Bortolo, Vicenza, Italy; 5 Neonatal Intensive Care Unit, Azienda Ospedaliero-Universitaria Policlinico - Vittorio Emanuele of Catania, Catania, Italy; 6 Department of Pediatrics, Pediatric and Neonatal intensive Care Unit, Azienda Ospedaliera Universitaria Integrata, Verona, Italy; 7 Neonatal Intensive Care Unit, Department of Medical and Surgical Sciences, IRCCS AOUBO, University of Bologna, Bologna, Italy; 8 Neonatal Intensive Care Unit and Neonatal Accompaniment Unit, San Marco Hospital, Azienda Ospedaliero-Universitaria Policlinico “Rodolico-San Marco”, University of Catania, Catania, Italy; 9 Department of Maternal Fetal and Neonatal Medicine, C. Arrigo Children’s Hospital, Alessandria, Italy; 10 Department of Human Pathology in Adult and Developmental Age “Gaetano Barresi”, Neonatal and Paediatric Intensive Care Unit, University of Messina, Messina, Italy; 11 Division of Neonatology, S. Giovanni Calibita Hospital Fatebenefratelli, Isola Tiberina, Rome, Italy; 12 Neonatal Intensive Care Unit and High-Risk Follow up program, Cà Foncello Regional Hospital, Azienda ULSS 2 Marca Trevigiana of Treviso, Treviso, Italy; 13 Division of Neonatology, “V. Buzzi” Children’s Hospital – ASST-FBF-Sacco, Milan, Italy; 14 Division of Neonatology, Neonatal Intensive Care Unit, Azienda Ospedaliero-Universitaria, Foggia, Italy; 15 Neonatal Intensive Care Unit, Fondazione IRCCS Ca’ Granda Ospedale Maggiore Policlinico of Milan, Milan, Italy; 16 Department of Clinical Sciences and Community Health, University of Milan, Milan, 20122 Italy; 17 Division of Neonatology and Neonatal Intensive Care Unit, Ospedale Evangelico Betania of Naples, Naples, Italy; 18 Division of Neonatology and Neonatal Intensive Care Unit, Azienda Ospedaliero Universitaria of Cosenza, Cosenza, Italy; 19 Maternal and Pediatrics Department, Maggiore Hospital, Bologna, Italy; 20 Department of Pediatrics, Padua, Italy; 21 Division of Neonatology, Catholic University of Rome, Rome, Italy; 22 SC Epidemiologia Clinica, Istituto di Ricovero e Cura a Carattere Scientifico Ospedale Policlinico San Martino of Genova, Genoa, Italy; 23 Division of Neonatology, Careggi University Hospital, Largo Brambilla 3, Firenze, 50134 Italy

**Keywords:** Surfactant, Multiple dose, Respiratory distress syndrome, Preterm infants

## Abstract

**Background:**

The issue of retreatment with surfactant of infants with respiratory distress syndrome (RDS) has been poorly investigated. Our aim was to identify possible clinical predictors of the need for multiple doses of surfactant in a large cohort of very preterm infants.

**Methods:**

Data were analyzed from three previous studies on infants born between 25^+ 0^ and 31^+ 6^ weeks of gestation with RDS who were treated with surfactant.

**Results:**

We studied 448 infants. Among them 306 (68%) were treated with a single dose of surfactant and 142 (32%) were treated with multiple doses. Multivariable mixed effects logistic regression analysis showed that the odd of requiring multiple doses of surfactant was significantly lower in patients with higher gestational age (27–28 vs. 25–26 wks: OR 0.46, 95% C.l. 0.26–0.79; *≥*29 vs. 25–26 wks: OR 0.34, 95% C.l. 0.13–0.85; overall *P* = 0.013), while it increased in infants born to mothers with hypertensive disorders of pregnancy (OR 2.53, 95% C.l. 1.49–4.31; *P* < 0.001) and with hemodynamically significant PDA (OR 2.74, 95% C.l. 1.66–4.53, *P* < 0.001).

**Conclusions:**

Gestational age, hypertension in pregnancy, and hemodynamically significant PDA can predict the need for multiple doses of surfactant. Further investigation is needed to evaluate if these sub-groups of preterm infants represent specific phenotypes of RDS who deserve a peculiar surfactant treatment.

**Supplementary Information:**

The online version contains supplementary material available at 10.1186/s13052-024-01828-1.

## Background

The occurrence of respiratory distress syndrome (RDS) in very preterm (gestational age < 32 weeks of gestation) and extremely preterm (gestational age < 26 weeks of gestation) infants is very high, ranging from 60 to 90% [1]. The cornerstones of RDS management are surfactant treatment and artificial respiratory supports. In fact, previous meta-analyses demonstrated that surfactant treatment significantly decreased mortality and the risk of air-leak in preterm infants with RDS [2], while the combination of early nasal continuous positive airway pressure (NCPAP) [3] and surfactant treatment [4] decreased the need for mechanical ventilation and the incidence of bronchopulmonary dysplasia (BPD) [5–7].

Great effort was made for establishing the most effective threshold for surfactant administration in preterm infants with RDS [8]. Current recommendations suggest treatment with surfactant when FiO_2_ > 0.30 [9], but lung ultrasound score is becoming the first-choice tool in many centers due to its higher predictive accuracy in this decision-making process [10, 11]. However, this relevant debate only concerns the administration of the first dose of surfactant because, although it is the most studied drug in the preterm infant, the issue of retreatment with further doses of surfactant has been poorly investigated.

A meta-analysis of two earlier studies found that multiple doses of surfactant compared to single reduced the risk of pneumothorax with a trend towards a decrease in mortality [12], but these studies differ from the current clinical practice due to the patients’ limited antenatal prophylaxis and the fixed timing of surfactant treatment. Nevertheless, recent data from the Canadian Neonatal Network (CNN) has shown that 25% of infants with gestational age < 29 weeks received > 1 dose of surfactant and that these patients have a higher risk of an adverse outcome [13]. These findings were partially confirmed by Ferri et al., who found a surfactant retreatment rate of 15% in very low birth weight infants and a similar association with adverse outcomes in these patients [14]. Other authors investigated the risk factors for surfactant retreatment and found that infants with lower gestational age [15], birth weight [16], and those small for gestational age (SGA) [15, 17] or born to mothers with hypertensive pregnancy disorders [15] are at a higher risk of surfactant retreatment. However, the evidence regarding the retreatment with surfactant in preterm infants with RDS remains incomplete, due to the limited number of studies, contradictory results, and the single-center design of previous studies [15–17].

Based on these considerations, we planned this study with the aim of identifying possible clinical predictors of the need for multiple doses of surfactant in a large cohort of very preterm infants. To achieve this objective, we compared clinical characteristics of infants who were treated with a single dose of surfactant with those of infants who were treated with multiple doses of surfactant.

## Methods

### Study design

Our pooled analysis used data from the data set of three previous studies: data from infants enrolled in the SLI trial (*n* = 291) from October 1, 2011, to January 31, 2013 at 15 Italian sites [18]; data from infants enrolled in the NIPAL trial (*n* = 200) from 1 December, 2020, to 31 October, 2022 at 15 Italian sites [19]; and data from infants enrolled in a study on caffeine in the delivery room (*n* = 29) from September 2019 to June 2021 at the NICU of the Careggi University Hospital of Florence [20]. All studies were approved by pediatric ethics committees of Tuscany (ID 29/2011, ID 234/2019, and ID 58/2019, respectively) and carried out in accordance with the Declaration of Helsinki. Infants were enrolled after informed parental consent. Only data of infants who were treated with surfactant were analyzed.

### Patients

Infants included in the study were born between 25^+ 0^ and 31^+ 6^ weeks of gestation and were affected by RDS requiring respiratory support [18–20]. RDS was defined as the presence of clinical signs of respiratory distress, oxygen dependence during the first 24 h of life, consistent chest radiograph appearances (decreased lung air content, reticulogranular pattern of the lungs, and air bronchograms), and the exclusion of other causes of respiratory failure [21]. Exclusion criteria were the presence of major congenital malformations, chromosomal disorders, and inherited metabolic diseases.

### Respiratory management

Resuscitation in the delivery room was performed following the guidelines of the AAP/AAH [22]. Consistently, infants who needed respiratory support were assisted with nasal continuous airway pressure (NCPAP) when they autonomously breathed or with positive pressure ventilation (PPV) when their breathing was ineffective, or they were apneic or persistently bradycardic [22]. Mechanical ventilation was started if patients had a heart rate < 60 bpm despite proper positive pressure ventilation [22]. In the neonatal intensive care unit (NICU), noninvasive respiratory support was performed using NCPAP, bi-level NCPAP, or nasal intermittent positive pressure ventilation (NIPPV). Moreover, infants were started on MV when the pH was < 7.20 with PaCO_2_ > 65 mmHg, or PaO_2_ < 50 mmHg with FiO_2_ *≥* 0.50, after surfactant treatment, or if they had frequent episodes of apnea (> 4 episodes in 1 h, or > 2 episodes requiring bag-and-mask ventilation) despite adequate NCPAP (5–7 cmH_2_O) delivery and oxygenation. Mechanical ventilation was performed using patient triggered ventilation with or without volume guarantee or high frequency ventilation. Respiratory supports were set to maintain a PaCO_2_ of 55–65 mmHg and 88–95% SpO_2_.

Infants were treated with surfactant (Curosurf^®^, Chiesi Farmaceutici Spa, Parma, Italy: 200 mg/Kg) if they required MV or a FiO_2_ > 0.30 [19, 20] or *≥* 0.40 [18] was necessary to maintain an adequate SpO_2_. Additional doses of surfactant (100 mg/Kg) were given to infants at the discretion of the attending neonatologist. Less invasive surfactant administration (LISA) and INtubation-SURfactant‐Extubation (InSURE) procedure were both used to administer surfactant to non-intubated patients.

Infants were commonly treated with intravenous or oral caffeine (Peyona^®^; Chiesi Farmaceutici S.p.A.; loading dose 20 mg/kg, maintenance dose 5 mg/kg).

### Collected data

We collected the following data from each trial [18–20]: gestational age, birth weight, birth weight < 10th, sex, singleton or twin, need for noninvasive respiratory support and MV, occurrence of patent ductus arteriosus (PDA) requiring treatment, intraventricular hemorrhage (IVH) *≥* 3° grade [23], periventricular leukomalacia (PVL) [24], sepsis, retinopathy of prematurity (ROP) [25], necrotizing enterocolitis (NEC) [26], BPD [27], and mortality.

The diagnosis of PDA requiring pharmacological treatment was made by echocardiographic demonstration of a ductal left- to-right shunt, with a left atrium to aortic root ratio > 1.3 or a ductal size > 1.5 mm.

The examined maternal variables included antenatal steroid treatment, type of delivery, placental abruption, hypertensive disorders, prolonged premature rupture of membranes (pPROM) > 18 h, and clinical chorioamnionitis (defined as the presence of fever with one or more of the following: maternal leukocytosis > 15,000/ mm^3^, uterine tenderness, fetal tachycardia, or foul-smelling amniotic fluid).

### Primary and secondary outcomes

The primary outcome measure was the number of surfactant doses administered per patient, categorized as single dose versus multiple doses. Secondary outcomes were the main clinical events observed during the hospitalization reported above.

### Statistical analysis

Categorical baseline characteristics were summarized as frequencies and percentages. Continuous variables were reported as mean and standard deviation. The Wilcoxon rank-sum test was used to compare continuous variables. Odds ratios with 95% confidence limits were estimated according to univariate and multivariate mixed effects logistic regression models, considering the trial a random factor. The F test was used to quantify the statistical significance of all coefficients. Only variables with a P value equal to or less than 0.20 at the univariate analysis were included in the multivariate model. All statistical tests were two-sided, and P values < 0.05 were considered to be statistically significant. No adjustment for multiple comparisons was made. Statistical analyses were performed by LB using SAS version 9.4 (SAS Institute, Cary, NC).

## Results

**Fig. 1:**
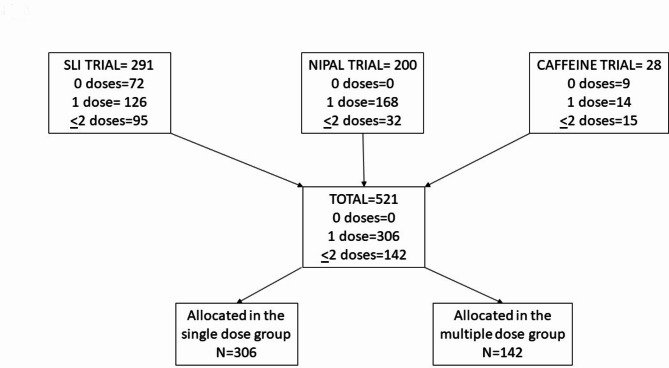
Flow chart of infants enrolled in the SLI trial [18], in the NIPAL trial [19] and in the study on caffeine in the delivery room [20] to perform the pooled data analysis

Data from 448 infants were analysed. Among them 306 (68%) were treated with a single dose of surfactant while 142 (32%) were treated with multiple doses. (Fig. 1) Infants who received multiple doses had lower gestational age (26.8 ± 1.5 vs. 28.0 ± 1.8 wks, *P* < 0.001) and lower birth weight (866 ± 272 vs. 1083 ± 319 g, *P* < 0.001) (Table 1).

**Table 1:** Baseline clinical characteristics of the infants and their mothers who received single or multiple doses of surfactant. Mean *±* (SD) or rate and (%)

Characteristics	Single dose (*n* = 306)	Multiple doses (*n* = 142)	*P*
**Infants**			
Gestational age (wks) 25–26 27–28 *≥*29	28.0 *±* 1.8 61 (20) 136 (44) 109 (36)	26.8 *±* 1.5 66 (47) 60 (23) 16 (12)	< 0.001
Birth weight (g) <10th percentile	1082 *±* 319 49 (16)	868 *±* 273 36 (39)	< 0.001 0.019
Female sex	149 (49)	62 (44)	0.321
Twins	112 (37)	44 (31)	0.246
**Mothers**			
Antenatal steroids	268 (88)	130 (92)	0.218
Cesarean section	260 (85)	121 (85)	0.947
Placental abruption	37 (12)	14 (10)	0.490
Hypertensive disorders of pregnancy	58 (19)	49 (35)	< 0.001
pPROM*	58 (19)	28 (20)	0.848
Chorioamnionitis	20 (7)	13 (9)	0.326

At the univariate analysis the administration of multiple doses of surfactant was significantly associated, among possible predictors, with lower gestational age, lower birth weight, birth weight < 10th percentile, PDA, and birth to mother with hypertensive disorders of pregnancy (Table 2).

**Table 2:** Univariate analysis of the association between potential clinical predictors and number of surfactant doses administered per patient. Rate and (%)

Variable	Total (*N* = 448)	Single dose (*N* = 306)	Multiple doses (*N* = 142)	Odds ratio	95% CI	*P* value*
Infants						
Sex						0.234
Male	237	157 (66)	80 (34)	1 (ref.)		
Female	211	149 (71)	62 (29)	0.78	0.51–1.18	
Gestational age (wks)						< 0.001
25–26	127	61 (48)	66 (52)	1 (ref.)		
27–28	196	136 (69)	60 (31)	0.43	0.27–0.69	
*≥* 29	125	109 (87)	16 (13)	0.24	0.11–0.50	
Birth weight						< 0.001
I tercile	150	76 (51)	74 (49)	1 (ref.)		
II tercile	150	107 (71)	43 (29)	0.43	0.26–0.71	
III tercile	148	123 (83)	25 (17)	0.33	0.19–0.60	
Small for gestational age						0.078
No	363	257 (71)	106 (29)	1 (ref.)		
Yes	85	49 (58)	36 (42)	1.59	0.95–2.65	
Twin pregnancy						0.531
No	292	194 (66)	98 (34)	1 (ref.)		
Yes	156	112 (72)	44 (28)	0.87	0.56–1.36	
Patent ductus arteriosus						< 0.001
No	235	192 (82)	43 (18)	1 (ref.)		
Yes	213	114 (54)	99 (46)	2.81	1.78–4.45	
Mothers						
Antenatal steroids						0.356
No	50	38 (76)	12 (24)	1 (ref.)		
Yes	398	268 (67)	130 (33)	1.40	0.69–2.86	
Type of delivery						0.660
Vaginal	67	46 (69)	21 (31)	1 (ref.)		
Cesarean	381	260 (68)	121 (32)	1.14	0.63–2.06	
Hypertensive disorders of pregnancy						< 0.001
No	339	249 (73)	90 (27)	1 (ref.)		
Yes	109	57 (52)	109 (48)	2.31	1.45–3.70	
Placental abruption						0.145
No	397	269 (68)	128 (32)	1 (ref.)		
Yes	51	37 (73)	14 (27)	0.61	0.31–1.19	
pPROM						0.250
No	362	248 (69)	114 (31)	1 (ref.)		
Yes	86	58 (67)	28 (33)	0.73	0.43–1.25	
Chorioamnionitis						0.767
No	415	286 (69)	129 (31)	1 (ref.)		
Yes	33	20 (61)	13 (39)	1.12	0.53–2.40	

*Mixed effects logistic regression model F test

Abbreviations: CI, confidence interval; ref., reference value; pPROM, prolonged premature rupture of membranes

Multivariable mixed effects logistic regression analysis showed that the odd of requiring multiple doses of surfactant was significantly lower in patients with higher gestational age (27–28 vs. 25–26 wks: OR 0.46, 95% C.l. 0.26–0.79; *≥*29 vs. 25–26 wks: OR 0.34, 95% C.l. 0.13–0.85; overall *P* = 0.013), while it increased in infants born to mothers with hypertensive disorders of pregnancy (OR 2.53, 95% C.l. 1.49–4.31; *P* < 0.001) and with hemodynamically significant PDA (OR 2.74, 95% C.l. 1.66–4.53, *P* < 0.001) increased it (Table 3).

**Table 3:** Multivariate analysis of the association between potential clinical predictors and number of surfactant doses administered per patient

	Odds ratio	95% CI	*P* value*
Gestational age (wks)			0.013
25–26	1 (ref.)		
27–28	0.46	0.26–0.79	
*≥* 29	0.34	0.13–0.85	
Birth weight			0.243
I tercile	1 (ref.)		
II tercile	0.61	0.33–1.11	
III tercile	0.80	0.36–1.78	
Small for age			0.982
No	1 (ref.)		
Yes	1.01	0.52–1.96	
Patent ductus arteriosus			< 0.001
No	1 (ref.)		
Yes	2.74	1.66–4.53	
Hypertensive disorders of pregnancy			< 0.001
No	1 (ref.)		
Yes	2.53	1.49–4.31	
Placental abruption			0.529
No	1 (ref.)		
Yes	0.79	0.39–1.63	

*Mixed effects logistic regression model F test

Abbreviations: CI, confidence interval; ref., reference value

An increased need for MV and occurrence of PDA, *≥* 3° grade IVH, BPD, ROP, late-onset sepsis, and mortality were significantly associated with the administration of multiple doses of surfactant (Table 4).

**Table 4:** Univariate analysis of the association between the number of surfactant doses administered per patient and the evaluated clinical outcomes. Rate and (%)

	Single dose (*N* = 306)	Multiple doses (*N* = 142)	Odds ratio	95% CI	*P* value*
Noninvasive mechanical ventilation	294 (96)	131 (92)	0.68	0.29–1.63	0.390
Mechanical ventilation	129 (42)	120 (85)	6.78	3.80–12.1	< 0.001
Grade 3–4 intraventricular haemorrhage	15 (5)	17 (12)	2.64	1.28–5.46	0.009
Bronchopulmonary dysplasia	101 (33)	86 (61)	2.76	1.78–4.28	< 0.001
Periventricular leukomalacia	6 (2)	5 (4)	1.84	0.55–6.15	0.324
Necrotizing enterocolitis	9 (3)	7 (5)	1.71	0.62–4.70	0.297
Retinopathy of prematurity	18 (6)	19 (13)	2.28	1.12–4.63	0.023
Early-onset sepsis	12 (4)	6 (4)	1.08	0.40–2.95	0.879
Late-onset sepsis	77 (25)	64 (45)	2.44	1.60–3.72	< 0.001
Death during hospitalization	20 (7)	24 (17)	2.91	1.55–5.48	0.001
Bronchopulmonary dysplasia and/or death	115 (38)	101 (71)	3.32	2.12–5.21	< 0.001

*Mixed effects logistic regression model F test

Abbreviations: CI, confidence interval

## Discussion

In this study we evaluated the need for surfactant retreatment in a large cohort of very preterm infants with the aim of identifying possible clinical predictors of surfactant redosing. We found that lower gestational age, hypertensive disorders of pregnancy, and PDA predicted the need for multiple doses of surfactant.

The effect of lower gestational age in increasing the surfactant retreatment rate was previously reported [13, 15, 17]. This finding is consistent with previous data demonstrating that in the animal model the synthesis of surfactant progressively increases during the last trimester of pregnancy [28], as also suggested by the simultaneous increase in lecithin/ sphingomyelin (L/S) ratio in the amniotic fluid [29]. On the other hand, the endogenous pool size of surfactant before treatment can vary largely in preterm infants with RDS ranging from 1 to 15 mg/kg [30]. Moreover, the effect of exogenous surfactant on endogenous synthesis seems to be associated in preterm infants with an increase in its synthesis as well its turnover [31]. These different aspects of surfactant metabolism can contribute to explain the subjective differences of need for single or multiple doses of surfactant, but with the common denominator that the more immature the lungs, the more vulnerable they are to ventilation-induced lung injury (VILI), oxidative stress, and inflammation and this can explain the lower response to the first dose of surfactant.

In agreement with Coshal [13] and Lanciotti [15], we found that hypertensive disorders of pregnancy increase the probability of preterm infants with RDS requiring retreatment with surfactant. This result is expected since hypertensive disorders of pregnancy were found to be correlated with a higher risk of severe RDS [31]. This correlation has been at least partially explained by a low level of the vascular endothelial growth factor (VEGF) and high level of the anti-angiogenic factors found in the cord blood of preterm infants born to mothers with hypertensive disorders of pregnancy which could negatively affect alveolarization and pulmonary vessels development [32]. Moreover, it has been observed in the animal model that high levels of VEGF are associated with an increased synthesis of surfactant [33] suggesting that an antenatal exposure to an anti-VEGF environment, as occurs in infants born to mothers with hypertensive disorders of pregnancy, could reduce the lung surfactant pool size leading to the need for multiple doses as shown by our study.

We found for the first time that PDA requiring treatment is an independent risk factor for multiple treatment with surfactant. Previous studies have rarely explored this possible correlation [13, 14, 17] and those that did only found an association which was not confirmed by logistic regression analysis [15]. These different results could be due to the different characteristics of studied populations, a different amount of the first dose of surfactant (100 or 200 ^15^ vs. 200 mg/kg), and possible different criteria for the diagnosis and management of PDA. However, our finding is in agreement with Beauchene et al., who recently reported that the status of PDA significantly affects the response to late (> 6 days of life) surfactant treatment which they mostly gave as retreatment in extremely preterm infants with persisting severe RDS [34]. On the other hand, it is well known that a hemodynamically significant PDA can lead to pulmonary fluid overload and subsequent pulmonary edema which, in turn, can induce surfactant inactivation [35]. This is caused by capillary-alveolar leakage of plasma components that interfere with the formation of the alveolar monolayer and impair functioning in a formed monolayer [35]. In fact, all soluble proteins, such as fibrinogen, hemoglobin, and albumin have been found to interfere with surfactant phospholipid de novo adsorption, since phospholipids adsorb more slowly than small amphiphilic proteins, which adsorb rapidly by molecular diffusion [35, 36].

We found that multiple surfactant doses were given more frequently to SGA infants than to no-SGA infants, but multivariate logistic regression analysis did not confirm this correlation in contrast with previous findings by Lanciotti et al. [15]. This different result might depend on the different characteristics of the study population (in our study the frequency of SGA was higher) and study design (multicenter vs. single-center [15] study; 200 vs. 100 mg/kg as initial dose of surfactant in about 1/3rd of patients [15]). In addition, in our study cohort, SGA infants more frequently had a lower birth weight and maternal hypertensive disorders of pregnancy. Moreover, among non-SGA infants, multiple doses of surfactant were strongly associated with lower birth weight and maternal hypertensive disorders of pregnancy, indicating the presence of population-based confounding factors (data not shown). However, since previous studies in animal model showed contradictory results [37, 38], we believe that further studies on the possible correlation between SGA condition in preterm infants and the need for multiple doses of surfactant would be useful.

The strength of our study includes the large size of our population, its multicenter design that supports the generalizability of findings, and the rigorousness of statistical analysis which supports the accuracy of results. A limitation of our study is that it represents a secondary analysis of data from three previous study data sets [18–20]. This precluded the possibility of evaluating important variables, such as severity of RDS, timing of PDA diagnosis and surfactant administration, mode of surfactant delivery (LISA or INSURE), and clinical response of treated patients.

## Conclusions

We found that the need for multiple doses of surfactant is higher in more immature infants, in infants born to mothers with hypertensive disorders of pregnancy, and in infants with PDA requiring treatment, most likely because of more severe RDS. Currently, it is not possible to know if it depends on decreased surfactant synthesis, increased turnover and inactivation, or the combination of these mechanisms. Further investigation is needed to evaluate if these sub-groups of preterm infants represent specific phenotypes of RDS who deserve a peculiar surfactant treatment.

## Electronic supplementary material

Below is the link to the electronic supplementary material.

Supplementary Material 1

## Data Availability

The datasets used and/or analysed during the current study are available from the corresponding author on reasonable request.

## Abbreviations

BPD: bronchopulmonary dysplasia
InSURE: INtubation-SURfactant‐Extubation
IVH: intraventricular hemorrhage
LISA: less invasive surfactant administration
NCPAP: nasal continuous positive airway pressure
NIPPV: nasal intermittent positive pressure ventilation
NEC: necrotizing enterocolitis
PDA: patent ductus arteriosus
PVL: periventricular leukomalacia
pPROM: prolonged premature rupture of membranes
RDS: respiratory distress syndrome
ROP: retinopathy of prematurity
SGA: small for gestational age
VEGF: vascular endothelial growth factor
