# Supplementary material for: Clinical predictors for surfactant retreatment in preterm infants with respiratory distress syndrome: the results of a pooled analysis
Source: Ital J Pediatr. 2025 Jan 5;51:1. doi: 10.1186/s13052-024-01828-1 (PMC11702167; doi:10.1186/s13052-024-01828-1)
Supplement: Supplementary file 1 — Supplementary Material 1 [file 13052_2024_1828_MOESM1_ESM.docx]

To:

Prof. Giovanni Corsello,

Editor in Chief of the

Italian Journal of Pediatrics

Florence, June 27, 2024

I am pleased to submit the following Research Article to the Italian Journal of Pediatrics:

**CLINICAL PREDICTORS FOR SURFACTANT RETREATMENT IN PRETERM INFANTS WITH RESPIRATORY DISTRESS SYNDROME: THE RESULTS OF A POOLED ANALYSIS**

Corresponding author:

Prof. Dr. Carlo Dani

Careggi University Hospital of Florence

*50139 Florence, Italy*

[carlo.dani@unifi.it](mailto:carlo.dani@unifi.it)

**Dear Prof. Corsello,**

many preterm infants receive multiple doses of surfactant but the issue of retreatment with surfactant of infants with respiratory distress syndrome (RDS) has been poorly investigated. Therefore, the aim of this study was to identify possible clinical predictors of the need for multiple doses of surfactant in a large cohort of very preterm infants.

We believe that this study is of significant interest for neonatologists because our results have been comprehensively commented in light of previous studies on this topic.

The manuscript is being submitted only to *Pediatric Pulmonology*, and will not be submitted elsewhere, while under consideration. There are no prior publications or submissions with any overlapping information, including studies and patients.

Conflict of Interest Disclosures: Prof. Carlo Dani received honoraria from Chiesi Farmaceutici SpA and Vyaire Medical Inc. for scientific consultancy.

Role of Funder/Sponsor: The present study was performed without specific funds.

All authors are responsible for the reported research and have participated in the concept and design, analysis and interpretation of data, drafting or revising of the manuscript, and have approved the manuscript as submitted.

Yours Faithfully,

Prof. Dr. Carlo Dani
